# Emotional Modulation of Conflict Processing in the Affective Domain: Evidence from Event-related Potentials and Event-related Spectral Perturbation Analysis

**DOI:** 10.1038/srep31278

**Published:** 2016-08-11

**Authors:** Jianling Ma, Chang Liu, Xu Chen

**Affiliations:** 1 Chongqing University of Posts and Telecommunications, Chongqing, 400065, China; 2 Faculty of Psychology, Southwest University, Chongqing, 400715, China; 3 Yangtze Normal University, Chongqing, 408100, China

## Abstract

Previous studies have revealed the impact of emotion on conflict processing. The present study was conducted to investigate whether cognitive control in the affective domain is also affected by emotion. Emotional face-word and body-word Stroop tasks were explored and contrasted, and both behavioural and electrophysiological measures were recorded. Behavioural results showed that both tasks replicated previous robust interference effects. At the physiological level, the two tasks showed dissociable neural activity in the early attention and perception stages. It was also found that the face-word task evoked more pronounced N1 and P2 amplitudes than the body-word task. However, the two tasks evoked comparable N450 amplitudes. At later processing stages, positive slow potentials were modulated by target emotion and congruency. In addition, time-frequency analyses also revealed that the face-word task induced enhanced theta activity compared to the body-word task at both early and later stages of processing. The present findings provide support for the dual competition framework and suggest the dynamic modulation of emotion on cognitive control in the affective domain.

Cognitive control is regarded as a key function of humans, serving to maintain normal life and allow adaptation to a changing environment. Cognitive control specifically refers to the ability to switch flexibly between appropriate and prepotent actions when faced with conflicting action tendency^1^,^2^. Thus, conflict detection and resolution is the core of cognitive control, and has already been explored for decades. Based on the influential conflict monitoring theory^2^,^3^,^4^, once a conflict is detected, the cognitive system will boost control efficiency, aiming to resolve subsequent trials. Indeed, several imaging studies have found that activity in the dorsal anterior cingulate cortex is correlated with conflict detection, whereas activity in the dorsolateral prefrontal cortex is associated with pronounced cognitive control^2^,^3^,^5^,^6^.

Under various circumstances, salient emotional stimuli may gain priority access to the cognitive processing system, and consequently interfere with an individual’s engagement in goal-directed behaviour. The affective modulation of conflict processing has been investigated extensively^7^,^8^,^9^,^10^,^11^,^12^,^13^,^14^,^15^. One approach to investigating the affective modulation of conflict processing is to explore how emotion exerts influence over performance on a cognitive control task. For example, on the Stroop color-word trial, the interference effect was larger when preceded by a negative emotional picture compared to a positive emotional picture^16^. In another recent study, participants were presented with positive, negative, and neutral emotional sounds prior to a Stroop colour-word trial in separate blocks^15^. The results showed that response latency on the Stroop colour-word trial was delayed when preceded by negative sound compared to positive and neutral sounds. In contrast to these studies, which have explored the direct and explicit influence of emotion on conflict processing, other studies have attempted to probe the implicit effect of emotion on conflict processing^7^,^8^,^9^,^10^,^11^. For example, in an adapted Flanker task, participants were instructed to identify the colour of the ink of the centre word while ignoring two flanker words above or below, for words with negative, positive or neutral valence. Results from studies using this task have shown that both negative and positive words speed up conflict processing.

Although existing evidence reveals that emotions affect conflict processing, an important yet unresolved issue is whether emotion also influences cognitive control in the affective domain. For example, in the emotional Stroop test, which is adapted from the classic colour-word Stroop paradigm, participants are required to judge emotion as expressed by a facial or body expression in the presence of inhibitory influences from a simultaneously presented emotion-laden word^17^,^18^,^19^,^20^,^21^,^22^,^23^,^24^,^25^. Imaging evidence shows that resolution of emotional Stroop conflict is associated with activity in the rostral anterior cingulate cortex, and that resolution of emotional conflict occurs via top-down inhibition of the amygdala by the rostral cingulate cortex^17^,^18^. Similar behavioural and electrophysiological data have been obtained using an emotional face-word and body-word task. However, these results do not necessarily imply that emotion exerts an influence on conflict processing, due to the fact that the goals of such studies were to explore the behavioural and neural dynamics of emotional conflict processing rather than the emotional modulation of conflict processing. Moreover, emotional conflict processing and emotional modulation of conflict processing have never been studied simultaneously. A study that employs the same paradigm in one experiment and manipulates the target is needed to clarify the emotional modulation of conflict processing within the affective domain. To this end, in the present study, both the emotional face-word and body-word Stroop tasks were explored.

Imaging approaches provide the exact brain structure that is involved in conflict detection and resolution, whereas behavioural measures capture the results of multiple sequential sub-stage processing. Furthermore, the high time-resolution of event-related potentials (ERPs) would provide the fine detail of each individual sub-stage processing. To date, two main ERP components have been reported for emotional Stroop tasks: the N450, which is related to conflict resolution, and a later stage slow potential^14^,^19^,^20^,^21^,^22^,^25^,^26^. The conflict N450 is a negative deflection in the time-locked ERP with frontal-central scalp distribution peaking around 400–500 ms after stimulus onset and index conflict detection, whereas the slow potential has a central-parietal scalp distribution at a later processing stage, which might be associated with conflict resolution^27^. It is plausible that cognitive control processing involves multiple successive stages, from early emotional target perception to later stage conflict detection and resolution. In the early stages, N1 and P2 are reliable neural markers of emotion and attention-related and perceptual processing^28^,^29^,^30^,^31^. Several studies have provided evidence that N1 and P2 can be modulated by the threat level of emotional stimuli. For example, high-threat stimuli evoked higher N1 and P2 amplitudes than low-threat stimuli^32^,^33^,^34^,^35^. With respect to the threat level of facial and body expressions, there is evidence that facial expressions are more threatening than body expressions, because facial expressions lead to more pronounced activity in the amygdala than threatening body expressions^36^,^37^,^38^,^39^. Based on these findings, it is possible that threatening facial expressions would evoke larger N1 and P2 amplitudes than body expressions in the early emotion perception stage. However, in the early stage where high cognitive control processing is not accessible, these two components may not be modulated by emotional congruency.

The onset of a particular type of stimulus can also induce event-related spectral perturbations (ERSPs) and oscillatory activity. Numerous prior cognitive control studies^40^,^41^,^42^,^43^,^44^,^45^,^46^,^47^ have found that specific bands of ERSP activity, such as increased theta activity, may be associated with a greater need for executive control under conflictive conditions. Our previous investigation^19^ also found augmented theta oscillations for the incongruent but not congruent condition in an emotional body-word task. We speculate that resolution of emotional conflict under the face-word task may also entail enhanced theta activity.

In summary, the purpose of the present study was to test whether the affective nature of the target influences emotional conflict processing. Based on the findings of our previous study, our hypothesis is as follows: (i) Longer response latencies and more errors would be found under the incongruent condition in both tasks, (ii) N1 and P2 would be modulated by stimuli type instead of emotional congruency, (iii) N450 and slow potentials (SP) would be modulated by emotional congruency rather than stimuli type, and (iv) enhanced theta oscillation activity underlying conflictive conditions for both tasks would be observed.

## Methods

### Participants

Twenty-seven undergraduate students took part in the formal ERP study. All participants were right-handed, had normal or corrected-to-normal vision, and had no history of psychiatric illness or neurological problems. Handedness was assessed through the Edinburgh Handedness Inventory. Furthermore, none of these subjects had participated previously in a similar ERP study. In this study, five participants were eliminated due to chance level of behavioural performance. The remaining participants were aged 18 to 26 years with a mean age of 23.6 years. The study was approved by the local Southwest University ethics committee. In accordance with the approved guidelines, written informed consent was obtained from the participants prior to conducting pilot or formal experiments. All methods were carried out in accordance with the approved guidelines.

### Materials and Procedure

In order to equate the body-word and face-word Stroop tasks, both tasks included negative angry and sad stimuli only. The body-word stimuli used in this study were identical to those used in our previous study. All body stimuli from the Bodily Expression Action Stimulus Test were subjected to a 4000-s forced-choice judgment. The resulting data showed that angry and sad bodies are better recognized than happy and fearful bodies. To minimize the influence of accuracy on behavioural data, 20 angry and 20 sad body-expression images of statistically equivalent accuracy were selected. These body images were compounded with Chinese words using Photoshop. The two Chinese characters, “
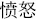
” (“angry”) and “
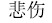
” (“sad”), were superimposed across the body in red colour in 28-point Times New Roman font. The position of the word was fixed on the chest of the body in all the body-word compound stimuli. A total of 80 compound images, in which 20 images for each of the four possible combinations of body-emotion and word-emotion, were generated. The four stimulus types (angry body-angry word, angry body-sad word, sad body-angry word, and sad body-sad word) then were divided into congruent (body expression matched emotional word) or incongruent (body expression did not match emotional word) categories. The incongruent category was expected to induce conflict in emotional conflict processing. For the face-word Stroop task, the stimuli consisted of 10 pictures of angry faces (5 male, 5 female) and 10 pictures of sad faces (5 male, 5 female) selected from the Chinese affective picture system. The two Chinese characters, “
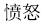
” (“angry”) and “
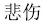
” (happy), were superimposed on the faces in red colour in 28-point Times New Roman font. The words and facial expressions were either congruent (e.g.,“
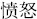
” superimposed on an angry face picture) or incongruent (e.g.,“
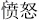
” superimposed on a happy face picture). Following ERP measurements, participants were required to rate the angry and sad expressions in terms of arousal and pleasantness using a 9-point scale: 1 (most pleasant) to 9 (most unpleasant); arousal: 1 (least arousing) to 9 (most arousing). Results showed that angry expressions (5.15 ± 0.21) were more arousing than sad expressions (4.68 ± 0.23), *F* (1, 21) = 8.346, *p* < 0.01, 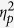 = 0.284; and body expressions (5.39 ± 0.24) were more arousing than facial expressions (4.44 ± 0.30), *F* (1, 21) = 6.660, *p* < 0.05, 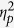 = 0.241. Angry expressions (3.13 ± 0.17) were not significantly more or less pleasant than sad expressions (3.09 ± 0.17), *F* (1, 21) = 0.073, *p* = 0.789; body expressions (3.4 ± 0.27) also were not significantly more or less pleasant than facial expressions (2.8 ± 0.27), *F* (1, 21) = 2.141, *p* = 0.158.

All compound stimuli were programmed and presented using E-Prime tools on a Dell 19-inch monitor. Participants were seated in a quiet room with dim light at a distance of 80 cm from the screen. The viewing angle of the framed body-word compound stimuli on the screen extended 9.87° vertically and 6.58° horizontally. Participants completed the body-word and face-word emotional Stroop tasks in two separate sessions. Participants were instructed to identify the emotion of the body or facial expression and ignore the meaning of the superimposed word. Session order was counterbalanced across participants. Subjects were instructed to respond as quickly and accurately as possible by pressing either “f” or “j” on the keyboard to indicate the expression of the image. Key allocations were counterbalanced across participants. All 80 compound body-word and 80 face-word stimuli were presented thrice and equally separated into 3 blocks, generating 240 trials in total for each task. Each block consisted of an equal number of congruent and incongruent trials. In each block, a trial began with a 500-ms fixation display, which was followed by a blank screen with a blank display that ranged from 300 to 600 ms. Then, the target body-word (or face-word) compound stimulus appeared at the centre of the screen for 1000 ms; participants were required to respond within this time window. The inter-trial interval display following each stimulus presentation was set randomly between 1200 and 1800 ms. Participants completed 20 practice trials before the start of each session. Participants were instructed to avoid blinking and other eye movements.

### EEG recording

Electroencephalography (EEG) was recorded from each participant at 64 sites on the scalp using Ag/AgCl electrodes mounted on an elastic cap (Brain Products GmbH, Gilching, Germany) with on-line references to the FCz electrode site. Vertical and horizontal electro-oculograms were recorded using electrodes placed below the right eye and from the right orbital rim, respectively. All inter-electrode impedances were maintained below 5 kΩ. Signals were amplified using a 0.01–100 Hz band-pass filter and continuously sampled at 500 Hz/channel.

### EEG analysis

Time-domain analysis included pre-processing using EEGLAB (Version 12.0.2.6b), an open toolbox running under the MATLAB environment. For time-domain analysis, all EEG signals were re-referenced off-line to TP9 and TP10 (average mastoid reference). An artifact subspace reconstruction algorithm was performed on continuous data to remove flat-line channels, eye-movement-related activity, low-frequency drifts, noisy channels, short-time bursts, and incompletely repaired segments from the data. Then, pre-processed clean EEG data were band-pass filtered to a range of 0.05 to 40 Hz, and epoched into a period of 1200 ms (200 ms baseline and 1000 ms post-stimulus onset). Signals were averaged across trials and time-locked to the onset of the compound stimuli separately for the congruent and incongruent categories for each task. Trials associated with erroneous responses and trials with a peak-to-peak deflection exceeding ±80 μV were excluded from averaging in both tasks. More than 80 and 100 trials were available for each subject for the face-word and body-word task analyses, respectively.

To assess dynamic changes in spectral power over time, raw EEG data were analysed in a similar manner to the ERP data as described above, except that data were band-pass filtered to a range of 1 to 40 Hz and epoched into a period of 1800 ms (600 ms pre-stimulus onset baseline and 1200 ms post-stimulus onset). To assess dynamic changes in spectral power over time, ERSP data were estimated and calculated using the *newtime*() function embedded in the EEGLAB toolbox. A Hanning tapered zero-padded fast Fourier transform (FFT) was applied to 1800-ms epoch data (900 frames per epoch). The window size of the moving FFT was set to 256 ms. For each condition (face-word congruent, face-word incongruent, body-word congruent, and body-word incongruent), the resulting estimated ERSP values were averaged across all trials and then converted to log power in decibels (dB). The baseline-normalized ERSP was obtained by subtracting the mean baseline (−400 ms to −100 ms pre-stimulus onset) power spectrum from each estimated spectrum, resulting in a 20 × 200 two-dimensional matrix representing time frequency for each electrode site and condition (20 frequencies from 0.97 to 31 Hz at each of 200 time points from −471 ms pre-stimulus onset to 1069 ms post-stimulus onset).

ERSP modulation was estimated in the following four steps:

(1) Based on our previous research, theta oscillation was more pronounced over the frontal midline area. We then plotted theta activity as a function of the post-stimulus onset time at Fz, F1, F2, F3, F4, FCz, FC1, FC2, FC3, and FC4.

(2) Judging from the time courses of theta power in the present study, the theta oscillation appeared to be more pronounced over two time windows: the early time window (0 to 300 ms) and the late (400 to 800 ms) time window. Based on these observations, we calculated the topographical distribution averaged across participants for theta activity in the intervals 0 to 300 ms and 400 to 800 ms, and confirmed that the enhancement of theta activity indeed originated in the frontal and frontal-central areas.

(3) The mass-univariate approach implemented in the *statcond* function of the EEGLAB toolbox was used to identify the time window in the ERSP values in which theta power was significantly different between the face-word and body-word tasks or between the incongruent and congruent conditions. A paired *t*-test was conducted on averaged time-frequency points including Fz, F1, F2, F3, F4, FCz, FC1, FC2, FC3, FC4, Cz, C1, C2, C3, and C4. For multiple comparisons, *P* < 0.05 was used to establish preliminary results of interest to prevent a too-conservative result from our false discovery rate (FDR) correction procedure. For theta band activity (3.9 to 7.8 Hz), the early time window (0–300 ms) and time points from 400 to 800 ms post-stimulus-onset were analysed.

(4) The resulting data showed that mean theta oscillations between 0 and 300 ms were significantly different between the face-word and body-word tasks. The mean theta oscillation between 450 and 750 ms was significantly different between congruent and incongruent conditions as well as between the face-word and body-word tasks at Fz, F1, F2, F3, FCz, FC1, FC2, and FC3. Because theta activity was more pronounced over the midline, only the traces from Fz, F1, F2, FCz, FC1, and FC2 were selected for further analysis.

### Behavioural data analysis

Reaction time and accuracy of the behavioural response were analysed with a 2 (task type: body-word vs. face-word) ×2 (congruency: congruent vs. incongruent) repeated-measures analysis of variance (ANOVA).

### ERP and ERSP data statistics

For ERP data analysis, the peak (baseline to peak) and latency of N1 (80–130 ms) and P2 (140–230 ms) were analysed at the following electrodes: F1, Fz, F2, FC1, FCz, and FC2. The peak (baseline to peak) and latency of N1 and P2 were subjected to a 2 (task type: body-word vs. face-word) ×2 (congruency: congruent vs. incongruent) ×6 (electrode site: F1, F2, Fz, FC1, FC2, and FCz) repeated-measures ANOVA. The mean amplitude within 400–550 ms of the N450 component was analysed at nine sites: F1, Fz, F2, C1, Cz, C2, CP1, CPz, and CP2. These nine electrodes were divided into two factors: area (frontal, central, and central-parietal) and hemisphere (left, middle, right). In the case of N450 data, a 2 (task type: body-word vs. face-word) ×2 (congruency: congruent vs. incongruent) ×3 (area: frontal, central, and central-parietal) ×3 (hemisphere: left, middle, and right) repeated-measures ANOVA was conducted. The mean amplitude within 580–700 ms of slow potentials was analysed at nine sites: FC1, FCz, FC2, C1, CZ, C2, CP1, CPz, and CP2. These nine electrodes were divided into two factors: area (frontal-central, central, and central-parietal) and hemisphere (left, middle, right). For slow potentials data, a 2 (task type: body-word vs. face-word) ×2 (congruency: congruent vs. incongruent) ×3 (area: frontal-central, central, and central-parietal) ×3 (hemisphere: left, middle, and right) repeated-measures ANOVA was conducted.

Theta-range ERSP values were averaged over the frequency from 3.9 to 7.8 Hz for the two time windows (early time window: 0 to 300 ms and the late time window: 450 to 750 ms). The resulting ERSP values were then entered into a three-way repeated-factor ANOVA: 2 (task type: body-word task vs. face-word task) × 2 (congruency: congruent vs. incongruent) ×electrode site (six levels: Fz, F1, F2, FCz, FC1, FC2) for the early (0–300 ms) and late (450–750 ms) time windows. All statistical analyses were performed using SPSS. Statistics were adjusted using the Greenhouse-Geisser epsilon correction for non-sphericity if the number of factor levels exceeded two. Uncorrected degrees of freedom and corrected *p*-values were reported. If applicable, main effects were followed by post hoc pairwise comparison with Bonferroni correction.

## Results

### Behavioural results

**Figure 1:**
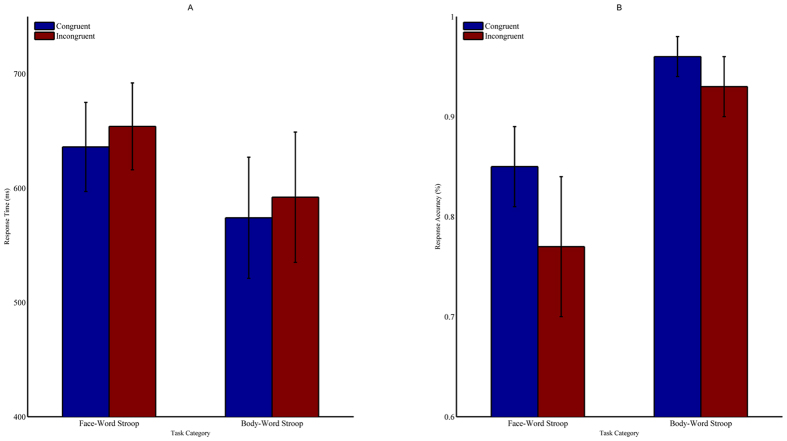
Behavioural responses on the emotional Stroop tasks. Response time (**A**) and response accuracy (**B**) results for the face-word and body-word Stroop tasks. Data are represented as the mean and standard deviation.

In the accuracy analysis, a significant congruency effect [*F* (1, 21) = 38.93, *p* < 0.001, 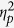 = 0.65], indicated by higher accuracy for congruent trials (90.4 ± 0.5%) compared to incongruent trials (84.8 ± 0.9%), was observed. The main effect of task type was significant [*F* (1, 21) = 128, *p* < 0.001, 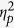 = 0.86], demonstrating that the body-word Stroop task (94.4 ± 0.5%) had a higher accuracy than the face-word Stroop task (80.8 ± 1.1%). The interaction between congruency and task was significant [*F* (1, 21) = 11.992, *p* < 0.01, 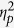 = 0.363]. The follow-up simple-effects test showed performance was better on the body-word task than the face-word task under both congruent and incongruent conditions (congruent condition: 95.7 ± 0.5% vs. 85 ± 1%, *F* (1, 21) = 82.51, *p* < 0.001; incongruent condition: 93 ± 0.7% vs. 76.5 ± 1.6%, *F* (1, 21) = 94.75, *p* < 0.001). Moreover, response time was faster on congruent trials (605 ± 9 ms) compared to incongruent trials (622 ± 8 ms, *F* (1, 21) = 16.63, *p* < 0.01, 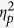 = 0.442) for trials with accurate responses. In addition, participants responded more quickly to trials on the body-word task (582 ± 11 ms) than to trials on the face-word task (645 ± 7 ms) *(F* (1, 21) = 42.35, *p* < 0.001, 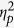 = 0.668) (Fig. 1).

### ERP results N1 effect

Analysis of the N1 amplitude revealed that the main effect of task type and congruency was significant [*F* (1, 21) = 58.46, *p* < 0.001, 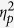 = 0.736; *F* (1, 21) = 30.535, *p* < 0.001, 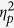 = 0.593]. The face-word task (−6.109 ± 0.44 μV) elicited a larger N1 amplitude than the body-word task (−3.508 ± 0.288 μV), and the incongruent condition (−5.172 ± 0.348 μV) elicited a more pronounced N1 amplitude than the congruent condition (−4.445 ± 0.331 μV). No other significant main effect or interaction was found. Analysis of the N1 latency revealed that the main effect of task type was significant [*F* (1, 21) = 28.899, *p* < 0.001, 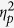 = 0.579]. The face-word task (102 ± 1.995 ms) elicited a shorter N1 latency than the body-word task (107 ± 1.901 ms). No other significant main effect or interaction was observed.

### P2 effect

**Figure 2:**
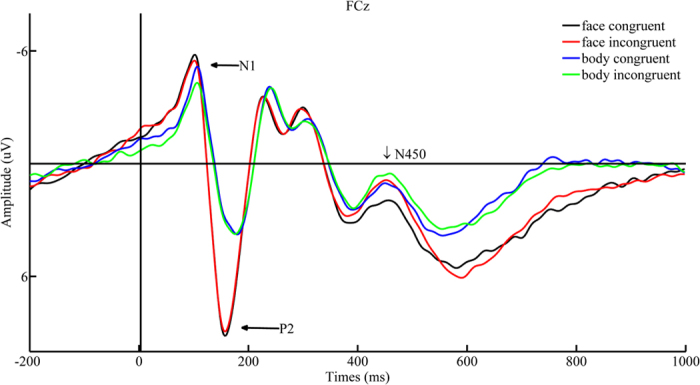
Grand-averaged waveforms in the frontal-central region. Grand-averaged waveforms at the “FCz” electrode site for face-congruent (black solid line), face-incongruent (red solid line), body-congruent (blue solid line), and body-incongruent (green solid line) trials as a function of time. The X-axis represents time within a trial, where 0 indicates the onset of the target stimulus. The Y-axis represents the wave amplitude range.

Analysis of the P2 amplitude revealed that the main effect of task type was significant [*F* (1, 21) = 51.62, *p* < 0.001, 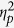 = 0.711]. The face-word task (10.39 ± 0.96 μV) elicited a larger P2 amplitude than the body-word task (5.32 ± 0.85 μV). No other significant main effect or interaction was observed. Analysis of the P2 latency revealed that the main effect of task type was significant [*F* (1, 21) = 19.559, *p* < 0.001, 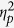 = 0.482]. The face-word task (159 ± 2.26 ms) elicited a faster P2 latency than the body-word task (172 ± 3.99 ms). No other main effect or interaction was found (Fig. 2).

### N450 effect

Analysis of the mean N450 amplitude revealed that the main effect of task type was not significant [*F* (1, 21) = 4.112, *p* = 0.055], but the main effect of congruency was significant [*F* (1, 21) = 12.535, *p* < 0.01, 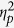 = 0.374]. The N450 amplitude corresponding to incongruent pairs (3.221 ± 0.538 μV) was more negative than that associated with congruent pairs (2.57 ± 0.553 μV). There was also a main effect of area [*F* (2, 42) = 42.936, *p* < 0.001, 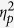 = 0.672], and post hoc Bonferroni comparison revealed that the N450 amplitude was more pronounced in the central-parietal area (3.97 ± 0.475 μV) than in the central (3.39 ± 0.58 μV) and frontal (1.328 ± 0.63 μV) areas. The main effect of hemisphere also was significant [*F* (2, 42) = 4.73, *p* < 0.05, 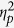 = 0.184]. Post hoc Bonferroni comparisons also revealed that the N450 amplitude was higher in the middle (3.078 ± 0.568 μV) than in the right (2.934 ± 0.544 μV) or left (2.678 ± 0.517 μV) hemispheres. No other significant effects were observed.

### Slow Potential effect

**Figure 3:**
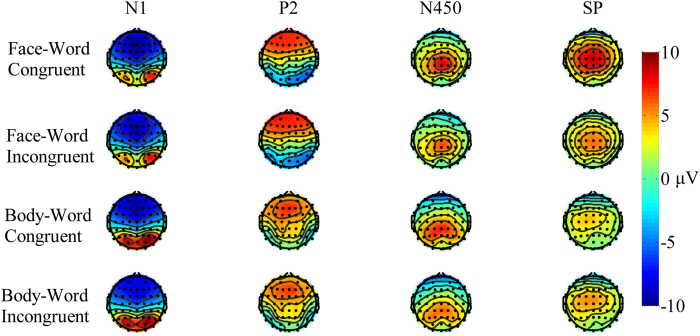
N1, P2, N450, and SP responses across tasks. Topographical distribution of N1 (80–130 ms), P2 (140–230 ms), N450 (400–550), and slow potentials (SP; 580–700 ms) averaged across participants for the face-congruent, face-incongruent, body-congruent, and body-incongruent conditions. Black dots represent scalp electrode positions. Contours connect points of equal amplitude on the waves.

Analysis of mean slow potential amplitude revealed a significant main effect of task type [*F* (1, 21) = 111.281, *p* < 0.01, 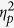 = 0.84]. The mean SP amplitude was more positive on the face-word task (4.73 ± 0.4 μV) than on the body-word task (2.01 ± 0.3 μV). The main effect of congruency was significant [*F* (1, 21) = 17.768, *p* < 0.05, 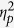 = 0.206]. The mean slow potential amplitude corresponding to incongruent pairs (3.2 ± 0.35 μV) was more positive than that associated with congruent pairs (3.5 ± 0.36 μV). No other significant effects were observed (Fig. 3).

### ERSP results

**Figure 4:**
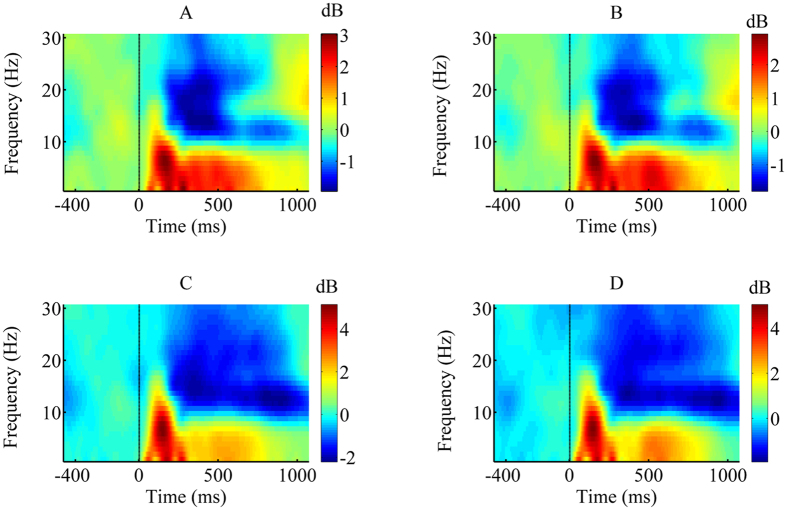
Grand-averaged time-frequency representation in the frontal-central region. Grand-averaged time-frequency representation (TF-R) plots at the “FCz” electrode site for face-congruent (**A**), face-incongruent (**B**), body-congruent (**C**), and body-incongruent (**D**) trials as a function of time. The X-axis represents time within a trial, where the dashed line indicates the onset of the target stimulus. The Y-axis represents the frequency range covered by our fast Fourier transform method.

**Figure 5:**
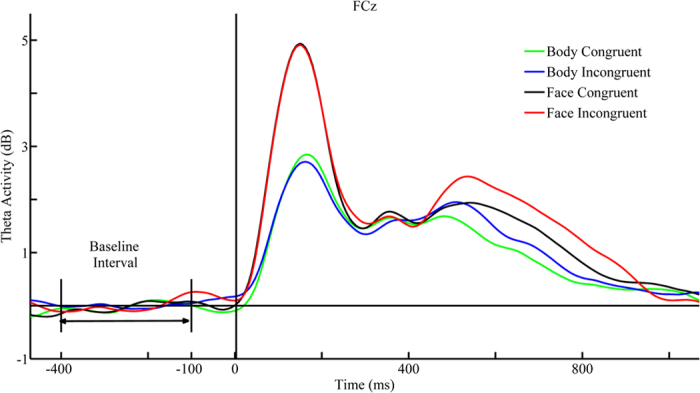
Theta activity time courses across tasks. Theta activity time courses extracted from the time-frequency representation (TF-R) at the FCz site for face-congruent (black solid line), face-incongruent (red solid line), body-congruent (green solid line), and body-incongruent (blue solid line) conditions. The X-axis represents time range transformed from our Fourier transform method within a trial, where the dashed line indicates the onset of the target stimulus. The Y-axis represents the theta power value covered by our fast Fourier transform method. The baseline-normalized theta event-related spectral perturbations (ERSP) was obtained by subtracting the mean baseline (−400-ms to −100-ms pre-stimulus onset) power.

Analysis of the early time window revealed that the main effect of task was statistically significant [*F* (1, 21) = 32.934, *p* < 0.001, 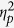 = 0.611]. The face-word task (2.34 ± 0.22 dB) induced a more boosted theta ERSP than the body-word task (1.55 ± 0.16 dB). No other significant effects were observed for the early time window. Analysis of the later time window revealed that the main effect of task type was significant [*F* (1, 21) = 10.148, *p* < 0.01, 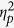 = 0.326]. The ERSP of the face-word task (1.483 ± 0.17 dB) was more enhanced than that associated with the body-word task (1.07 ± 0.08 dB). The main effect of congruency was significant [*F* (1, 21) = 8.35, *p* < 0.01, 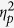 = 0.293]. The ERSP corresponding to incongruent pairs (1.403 ± 0.128 dB) was larger than that associated with congruent pairs (1.152 ± 0.12 dB). No other significant main effects or interactions were observed for the late time window (Figs 4 and 5).

## Discussion

The present study aimed to examine emotional modulation of cognitive control in the affective domain by exploring emotional face-word and body-word conflict tasks. Behaviourally, both tasks replicated previous interference effects, revealing both a slower response time and higher error rate for incongruent conditions than for congruent conditions and a stronger interference effect for the face-word task than for the body-word task. ERP results revealed both common and dissociable neural activities across the two tasks: the face-word task evoked more enhanced early and later stage neural activity than the body-word task as indexed by more pronounced N1, P2, and Slow potential amplitudes for the face-word task relative to the body-word task, whereas the two tasks evoked comparable N450 amplitudes. Moreover, time-frequency representation analysis revealed that the face-word task induced more boosted theta oscillation than the body-word task at early and late stages. Based on these findings, we concluded that cognitive control in the affective domain, at least as manifested by the emotional Stroop task, can be modulated by the target emotion.

Our behavioural data are consistent with previous studies reporting robust behavioural interference effects^14^,^17^,^18^,^19^,^20^,^22^,^25^,^48^. Interestingly, the present study revealed higher accuracy and faster response time on the body-word task than the face-word task. Such behavioural performance differences are more obvious at the neural level. We found that the two tasks evoked dissociable neural activity as measured by EEG, including larger N1 and P2 amplitudes and boosted theta oscillation during the face-word task compared to the body-word task. N1 and P2 are suggested as reliable neural markers of early emotional attention and perceptual processing, and are always generally coupled with selective augmented theta-band activities^40^,^44^,^45^,^49^,^50^. Our results are consistent with these previous studies. Moreover, such dissociable neural activities indicate that facial and body expressions evoked distinct responses despite both representing negative stimuli, which is consistent with prior evidence^36^,^37^,^38^,^39^, demonstrating that threatening facial and body expressions may evoke different brain activity.

Both tasks evoked a pronounced N450 amplitude under incongruent conditions, which is consistent with previous tasks performed in both the emotional and cognitive domains^26^,^50^,^51^,^52^,^53^,^54^. Although the underlying meaning of this component remains controversial, it is commonly believed that N450 reflects conflict detection processing^18^,^25^,^27^,^52^,^55^. Thus, it is possible that N450 might be correlated to the detection of emotional conflict between emotional target expression and the task irrelevant distractor in the present study. In addition, our finding that N450 amplitudes were not significantly different between the face-word task and body-word task suggests that may reflect conflict detection but not intentional control processing.

We found a significant slow potential congruency effect and theta effect. Slow potential may be associated with conflict resolution or post-response monitoring, and is believed to reflect voluntary control processing^53^,^56^,^57^,^58^. More importantly, Slow potential amplitudes were larger and theta activity was stronger during the face-word task than the body-word task. Furthermore, both tasks induced enhanced theta oscillation under the incongruent condition but not the congruent condition. If a larger amplitude reflects a more elaborate control process^59^,^60^ and theta oscillation reflects the need to control the distractor^41^,^46^,^47^,^61^, then together these two neural indexes may indicate that the face-word task was more difficult to execute, which is consistent with the inferior behavioural performance observed on the face-word task.

Based on the behavioural and neural observations, the results of the present study bolster the ‘dual-competition’ framework, which aims to explain how emotion interacts with executive control^16^,^62^,^63^. As proposed by this model, emotion (affective significance) directs cognitive control mainly through perceptual and executive competition. The threat level of the stimulus is the determinant of perceptual competition. Low-threat stimuli exert a modest effect whereas high-threat stimuli exert a deleterious effect on the perceptual stage. The results of the present study extend these findings by demonstrating a differential effect of the threat level on conceptual processing such that stimuli with higher levels of threat elicit stronger effects.Threatening stimuli draw more attention to common resources than non-threatening stimuli, thus leaving fewer resources dedicated to executive control processes, and consequently reducing the ability to control conflict^56^. Based on the behavioural findings in this study, negative facial expressions could be regarded as high-threat stimuli, whereas negative body expressions could be regarded as relatively low-threat stimuli. In a similar manner, fewer resources may be recruited during the presentation of threatening facial stimuli compared to threatening body stimuli. Consequently, more control effort, as evidenced by more pronounced later-stage SP amplitudes and theta oscillation in the face-word task, is needed to resolve the emotional conflict.

It should be noted that the proposed dual competition model was initially applicable to cognitive control context. Despite the fact that the context varies considerably, the shared feature is that the cognitive control task instead of the emotional task is the primary task, and the primary aim is to investigate how affective information exerts control on a pure cognitive control process^64^. However, our study and a recent study^54^ fall into another category in which participants are required to resolve the emotional conflict rather than the cognitive conflict. It is clear that the dual competition model could also be extended to the emotional conflict domain. Furthermore, the nature of the target emotion *per se* could have an effect on emotional conflict processing.

There remain unresolved issues in the current study. Although early and later stage brain activity was modulated by the target threat level, whether such modulation is of a quantitative or qualitative nature remains unknown. Are identical brain structures responsible for the resolution of both face-word and body-word conflict with variation only in the magnitude of activity, or are both common and dissociable brain areas involved in conflict resolution related to the face-word and body-word tasks? Another unresolved issue is whether this modulation effect would emerge in other tasks, such as the Flanker or Simon task. Further studies are needed to solve these issues. In summary, the present study provides evidence that emotional conflict processing is modulated by the target stimuli. Thus, our results address the importance of considering the nature of the target when exploring emotional conflict processing.

## Additional Information

**How to cite this article**: Ma, J. *et al*. Emotional Modulation of Conflict Processing in the Affective Domain: Evidence from Event-related Potentials and Event-related Spectral Perturbation Analysis. *Sci. Rep.* **6**, 31278; doi: 10.1038/srep31278 (2016).

## References

[b1] Botvinick M. & Braver T. Motivation and Cognitive Control: From Behavior to Neural Mechanism. Annu. Rev. Psych. 66, 83–113 (2014). 10.1146/annurev-psych-010814-015044 25251491

[b2] Botvinick M. M., Carter C. S., Braver T. S., Barch D. M. & Cohen J. D. Conflict Monitoring and Cognitive Control. Psychol. Rev. 108, 624–652 (2001). 11488380 10.1037/0033-295x.108.3.624

[b3] Botvinick M., Nystrom L. E., Fissell K., Carter C. S. & Cohen J. D. Conflict monitoring versus selection-for-action in anterior cingulate cortex. Nature 402, 179–181 (1999). 10647008 10.1038/46035

[b4] Botvinick M. M., Cohen J. D. & Carter C. S. Conflict monitoring and anterior cingulate cortex: an update. Trends Cogn. Sci. 8, 539–546 (2004). 15556023 10.1016/j.tics.2004.10.003

[b5] Egner T. & Hirsch J. Cognitive control mechanisms resolve conflict through cortical amplification of task-relevant information. Nat. Neurosci. 8, 1784–1790 (2005). 16286928 10.1038/nn1594

[b6] Cohen J. D., Botvinick M. & Carter C. S. Anterior cingulate and prefrontal cortex: who’s in control? Nat. Neurosci. 12, 421–423 (2000). 10769376 10.1038/74783

[b7] Kanske P. & Kotz S. A. Emotion speeds up conflict resolution: a new role for the ventral anterior cingulate cortex? Cereb. Cortex, 21, 911–919 (2010). 20732901 10.1093/cercor/bhq157

[b8] Kanske P. & Kotz S. A. Positive Emotion speeds up conflict processing: ERP response in an auditory Simon task? Biol. Psychol. 87, 122–127 (2011). 21382438 10.1016/j.biopsycho.2011.02.018

[b9] Kanske P. & Kotz S. A. Modulation of early conflict processing: N200 responses to emotional words in a flanker task. Neuropsychologia 48, 3661–3664 (2010). 20654636 10.1016/j.neuropsychologia.2010.07.021

[b10] Kanske P. & Kotz S. A. Emotion triggers executive attention: anterior cingulate cortex and amygdala responses to emotional words in a conflict task. Hum. Brain Mapp. 32, 198–208 (2011). 20715084 10.1002/hbm.21012 PMC6870409

[b11] Kanske P. & Kotz S. A. Conflict processing is modulated by positive emotion: ERP data from a flanker task. Behav. Brain Res. 219, 382–386 (2011). 21295076 10.1016/j.bbr.2011.01.043

[b12] Zinchenko A., Kanske P., Obermeier C., Schröger E. & Kotz S. A. Emotion and goal-directed behavior: ERP evidence on cognitive and emotional conflict. Soc. Cog. Affect. Neurosci. 10, 1577–1587 (2015). 10.1093/scan/nsv050 PMC4631156 25925271

[b13] Chiew K. S. & Braver T. S. Dissociable influences of reward motivation and positive emotion on cognitive control. Cogn. Affect. Behav. Neurosci. 14, 509–529 (2014). 24733296 10.3758/s13415-014-0280-0 PMC4072919

[b14] Xue S. . Positive emotion modulates cognitive control: An event‐related potentials study. Scand. J. Psychol. 54, 82–88 (2013). 23397988 10.1111/sjop.12031

[b15] Yuan J. . Pleasant mood intensifies brain processing of cognitive control: ERP correlates. Biol. Psychol. 87, 17–24 (2011). 21315134 10.1016/j.biopsycho.2011.01.004

[b16] Padmala S., Bauer A. & Pessoa L. Negative emotion impairs conflict-driven executive control. Front. Psychol. 2, 192 (2011). 21886635 10.3389/fpsyg.2011.00192 PMC3154405

[b17] Egner T., Etkin A., Gale S. & Hirsch J. Dissociable neural systems resolve conflict from emotional versus nonemotional distracters. Cereb. Cortex 18, 1475–1484 (2008). 17940084 10.1093/cercor/bhm179

[b18] Etkin A., Egner T., Peraza D. M., Kandel E. R. & Hirsch J. Resolving emotional conflict: a role for the rostral anterior cingulate cortex in modulating activity in the amygdala. Neuron 51, 871–882 (2006). 16982430 10.1016/j.neuron.2006.07.029

[b19] Ma J., Liu C. & Chen X. Emotional conflict processing induce boosted theta oscillation. Neurosci. Lett. 595, 69–73 (2015). 25863173 10.1016/j.neulet.2015.04.009

[b20] Ma J., Liu C., Zhong X., Wang L. & Chen X. Emotional Body-Word Conflict Evokes Enhanced N450 and Slow Potential. PloS ONE 9, e95198 (2014). 24819150 10.1371/journal.pone.0095198 PMC4018289

[b21] Xue S., Ren G., Kong X., Liu J. & Qiu J. Electrophysiological correlates related to the conflict adaptation effect in an emotional conflict task. Neurosci. Lett. 584, 219–223 (2014). 25459295 10.1016/j.neulet.2014.10.019

[b22] Zhu X. R., Zhang H. J., Wu T. T., Luo W. B. & Luo Y. J. Emotional conflict occurs at an early stage: Evidence from the emotional face–word Stroop task. Neurosci. Lett. 478, 1–4 (2010). 20417689 10.1016/j.neulet.2010.04.036

[b23] Marusak H. A., Martin K. R., Etkin A. & Thomason M. E. Childhood trauma exposure disrupts the automatic regulation of emotional processing. Neuropsychopharmacology 40, 1250–1258 (2015). 25413183 10.1038/npp.2014.311 PMC4367470

[b24] Deng Z. . Regional gray matter density associated with emotional conflict resolution: Evidence from voxel-based morphometry. Neuroscience 275, 500–507 (2014). 24976515 10.1016/j.neuroscience.2014.06.040

[b25] Shen Y., Xue S., Wang K. & Qiu J. Neural time course of emotional conflict control: An ERP study. Neurosci. Lett. 541, 34–38 (2013). 23454616 10.1016/j.neulet.2013.02.032

[b26] Xue S. . The dissociable neural dynamics of cognitive conflict and emotional conflict control: An ERP study. Neurosci. Lett. 619, 149–154 (2016). 26987720 10.1016/j.neulet.2016.03.020

[b27] Clawson A., Clayson P. E. & Larson M. J. Cognitive control adjustments and conflict adaptation in major depressive disorder. Psychophysiology 50, 711–721 (2013). 23735120 10.1111/psyp.12066

[b28] Delplanque S., Lavoie M. E., Hot P., Silvert L. & Sequeira H. Modulation of cognitive processing by emotional valence studied through event-related potentials in humans. Neurosci. Lett. 356, 1–4 (2004). 14746887 10.1016/j.neulet.2003.10.014

[b29] Cuthbert B. N., Schupp H. T., Bradley M. M., Birbaumer N. & Lang P. J. Brain potentials in affective picture processing: covariation with autonomic arousal and affective report. Biol. Psychol. 52, 95–111 (2000). 10699350 10.1016/s0301-0511(99)00044-7

[b30] Schupp H. T., Junghöfer M., Weike A. I. & Hamm A. O. Attention and emotion: an ERP analysis of facilitated emotional stimulus processing. Neuroreport 14, 1107–1110 (2003). 12821791 10.1097/00001756-200306110-00002

[b31] Olofsson J. K., Nordin S., Sequeira H. & Polich J. Affective picture processing: an integrative review of ERP findings. Biol. Psychol. 77, 247–265 (2008). 18164800 10.1016/j.biopsycho.2007.11.006 PMC2443061

[b32] Keil A. . Large‐scale neural correlates of affective picture processing. Psychophysiology 39, 641–649 (2002). 12236331 10.1017/S0048577202394162

[b33] Scott G. G., O’Donnell P. J., Leuthold H. & Sereno S. C. Early emotion word processing: Evidence from event-related potentials. Biol. Psychol. 80, 95–104 (2009). 18440691 10.1016/j.biopsycho.2008.03.010

[b34] Herbert C., Kissler J., Junghöfer M., Peyk P. & Rockstroh B. Processing of emotional adjectives: Evidence from startle EMG and ERPs. Psychophysiology 43, 197–206 (2006). 16712590 10.1111/j.1469-8986.2006.00385.x

[b35] Bar-Haim Y., Lamy D. & Glickman S. Attentional bias in anxiety: A behavioral and ERP study. Brain Cogn. 59, 11–22 (2005). 15919145 10.1016/j.bandc.2005.03.005

[b36] Kret M., Pichon S., Grèzes J. & de Gelder B. Similarities and differences in perceiving threat from dynamic faces and bodies. An fMRI study. Neuroimage 54, 1755–1762 (2011). 20723605 10.1016/j.neuroimage.2010.08.012

[b37] Kret M. E., Pichon S., Grèzes J. & De Gelder B. Men fear other men most: gender specific brain activations in perceiving threat from dynamic faces and bodies–an fMRI study. Front. Psychol. 2, 3 (2011). 21713131 10.3389/fpsyg.2011.00003 PMC3111446

[b38] Kret M. E., Stekelenburg J. J., Roelofs K. & De Gelder B. Perception of face and body expressions using electromyography, pupillometry and gaze measures. Front. Psychol 4, 28 (2013). 23403886 10.3389/fpsyg.2013.00028 PMC3567353

[b39] Pichon S., de Gelder B. & Grèzes J. Two different faces of threat. Comparing the neural systems for recognizing fear and anger in dynamic body expressions. Neuroimage 47, 1873–1883 (2009). 19371787 10.1016/j.neuroimage.2009.03.084

[b40] Bekkedal M. Y., Rossi J. & Panksepp J. Human brain EEG indices of emotions: delineating responses to affective vocalizations by measuring frontal theta event-related synchronization. Neurosci. Biobehav. Rev. 35, 1959–1970 (2011). 21596060 10.1016/j.neubiorev.2011.05.001

[b41] Cavanagh J. F. & Frank M. J. Frontal theta as a mechanism for cognitive control. Trends Cogn. Sci. 18, 414–421 (2014). 24835663 10.1016/j.tics.2014.04.012 PMC4112145

[b42] Cohen M. X. & Cavanagh J. F. Single-trial regression elucidates the role of prefrontal theta oscillations in response conflict. Front. Psychol. 2, 30 (2011). 21713190 10.3389/fpsyg.2011.00030 PMC3111011

[b43] Cohen M. X. & Donner T. H. Midfrontal conflict-related theta-band power reflects neural oscillations that predict behavior. J. Neurophysiol. 110, 2752–2763 (2013). 24068756 10.1152/jn.00479.2013

[b44] Knyazev G. G., Slobodskoj-Plusnin J. Y. & Bocharov A. V. Gender differences in implicit and explicit processing of emotional facial expressions as revealed by event-related theta synchronization. Emotion 10, 678 (2010). 21038950 10.1037/a0019175

[b45] Maratos F. A., Mogg K., Bradley B. P., Rippon G. & Senior C. Coarse threat images reveal theta oscillations in the amygdala: A magnetoencephalography study. Cogn. Affect. Behav. Neurosci. 9, 133–143 (2009). 19403890 10.3758/CABN.9.2.133

[b46] Nigbur R., Cohen M., Ridderinkhof K. & Stürmer B. Theta Dynamics Reveal Domain-specific Control over Stimulus and Response Conflict. J. Cogn. Neurosci. 24, 1264–1274 (2012). 21861681 10.1162/jocn_a_00128

[b47] Nigbur R., Ivanova G. & Stürmer B. Theta power as a marker for cognitive interference. Clin. Neurophysiol. 122, 2185–2194 (2011). 21550845 10.1016/j.clinph.2011.03.030

[b48] Egner T., Delano M. & Hirsch J. Separate conflict-specific cognitive control mechanisms in the human brain. Neuroimage 35, 940–948 (2007). 17276088 10.1016/j.neuroimage.2006.11.061

[b49] Sun J., Sun B., Wang B. & Gong H. The processing bias for threatening cues revealed by event-related potential and event-related oscillation analyses. Neuroscience 203, 91–98 (2012). 22233779 10.1016/j.neuroscience.2011.12.038

[b50] DeLaRosa B. L. . Electrophysiological spatiotemporal dynamics during implicit visual threat processing. Brain Cogn. 91, 54–61 (2014). 25222294 10.1016/j.bandc.2014.08.003

[b51] Szűcs D. & Soltész F. Functional definition of the N450 event-related brain potential marker of conflict processing: a numerical Stroop study. BMC Neurosci. 13, 35 (2012). 22452924 10.1186/1471-2202-13-35 PMC3383462

[b52] Clayson P. E., Larson M. J. & Astikainen P. S. Adaptation to emotional conflict: evidence from a novel face emotion paradigm. PloS ONE 8, e75776 (2013). 24073278 10.1371/journal.pone.0075776 PMC3779161

[b53] West R. Neural correlates of cognitive control and conflict detection in the Stroop and digit-location tasks. Neuropsychologia 41, 1122–1135 (2003). 12667546 10.1016/s0028-3932(02)00297-x

[b54] West R., Jakubek K., Wymbs N., Perry M. & Moore K. Neural correlates of conflict processing. Exp. Brain Res. 167, 38–48 (2005). 16082533 10.1007/s00221-005-2366-y

[b55] Larson M. J., Kaufman D. A. & Perlstein W. M. Neural time course of conflict adaptation effects on the Stroop task. Neuropsychologia 47, 663–670 (2009). 19071142 10.1016/j.neuropsychologia.2008.11.013

[b56] Inzlicht M., Bartholow B. D. & Hirsh J. B. Emotional foundations of cognitive control. Trends Cogn. Sci. 19, 126–132 (2015). 25659515 10.1016/j.tics.2015.01.004 PMC4348332

[b57] Liu Y., Wang Z., Quan S. & Li M. The effect of positive affect on conflict resolution: Modulated by approach-motivational intensity. Cogn. Emot. 11, 1–14 (2015). 10.1080/02699931.2015.1081874 26357903

[b58] Soutschek A., Müller H. J. & Schubert T. Conflict-specific effects of accessory stimuli on cognitive control in the Stroop task and the Simon task. Exp. Psychol. 60, 140–147 (2013). 23128585 10.1027/1618-3169/a000181

[b59] Vuilleumier P. How brains beware: neural mechanisms of emotional attention. Trends Cogn. Sci. 9, 585–594 (2005). 16289871 10.1016/j.tics.2005.10.011

[b60] Wendt M., Kluwe R. H. & Peters A. Sequential modulations of interference evoked by processing task-irrelevant stimulus features. J. Exp. Psychol. Hum. Percept. Perform. 32, 644–667 (2006). 16822130 10.1037/0096-1523.32.3.644

[b61] Cavanagh J. F., Cohen M. X. & Allen J. J. Prelude to and resolution of an error: EEG phase synchrony reveals cognitive control dynamics during action monitoring. J. Neurosci. 29, 98–105 (2009). 19129388 10.1523/JNEUROSCI.4137-08.2009 PMC2742325

[b62] Pessoa L. How do emotion and motivation direct executive control? Trends Cogn. Sci. 13, 160–166 (2009). 19285913 10.1016/j.tics.2009.01.006 PMC2773442

[b63] Lindström B. R. & Bohlin G. Threat-relevance impairs executive functions: negative impact on working memory and response inhibition. Emotion 12, 384–393 (2012). 22468619 10.1037/a0027305

[b64] Hart S. J., Green S. R., Casp M. & Belger A. Emotional priming effects during Stroop task performance. Neuroimage 49, 2662–2670 (2010). 19883772 10.1016/j.neuroimage.2009.10.076 PMC2818423

